# Ion-molecule reactions catalyzed by a single gold atom†

**DOI:** 10.1039/d0sc03523h

**Published:** 2020-07-27

**Authors:** Shengfu Yang, Hong Wu, Qiquan Luo, Aula M. Al Hindawi, Berlian Sitorus, Andrew M. Ellis, Jinlong Yang

**Affiliations:** School of Chemistry, University of Leicester Leicester LE1 7RH UK sfy1@le.ac.uk; Hefei National Laboratory for Physical Sciences at the Microscale, University of Science and Technology of China Hefei 230026 P. R. China jlyang@ustc.edu.cn; Department of Chemistry, College of Education for Pure Science, University of Karbala Kerbala Iraq; Department of Chemistry, Tanjungpura University Pontianak Indonesia

## Abstract

We report that Au atoms within van der Waals complexes serve as catalysts for the first time. This was observed in ionization-induced chemistry of 1,6-hexanediol–Au and 1,8-octanediol–Au complexes formed in superfluid helium nanodroplets, where the addition of Au atom(s) made C_2_H_4_^+^ the sole prominent product in dissociative reactions. Density functional theory (DFT) calculations showed that the Au atom significantly strengthens all of the C–C bonds and weakens the C–O bonds in the meantime, making the C–C bonds stronger than the two C–O bonds in the ionized complexes. This leads to a preferential cleavage of the C–O bonds and thus a strong catalytic effect of the Au atoms in the reactions.

## Abstract 2

Single Au atoms within van der Waals complexes are found to serve as catalysts in ionisation-induced chemistry for the first time.

## Graphical abstract

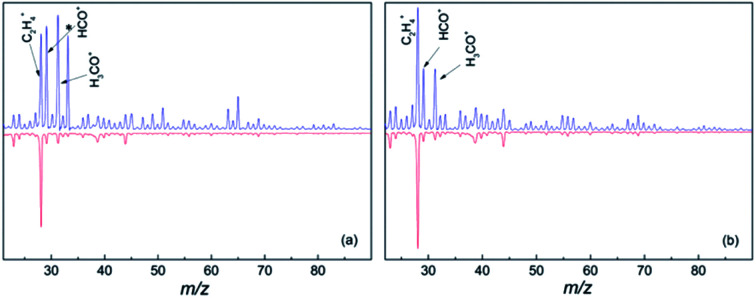

## Introduction

Single metal atoms anchored on a surface can have remarkably high catalytic activity and selectivity because of the large surface-to-volume ratios and/or modified catalytic properties.^1,2^ Recent discoveries include atomically dispersed Pt atoms and alkali ions on surfaces of alumina and silica that catalyze low-temperature water–gas shift reactions,^3^ single Pt atoms on metal oxide surfaces for CO oxidation,^4,5^ isolated Pd atoms on Cu(111) surface for selective hydrogenation of styrene and acetylene,^6^ crown-jewel-structured Pd/Au nanoparticles with isolated gold atoms as active sites for H_2_O_2_ decomposition,^7^ FeOx-supported single Pt atoms for hydrogenation of substituted nitroarenes,^8^ and atomically dispersed Fe(OH)_*x*_ on Pt surface for CO oxidation in hydrogen.^9^ However, in all of these studies the metal atoms are affixed to a surface, where their chemical and/or electronic properties are inevitably modified by the surface. To dissect the surface effect and explore the ultimate limit of catalysis at the molecular level, single atom catalysis has been extensively explored in the gas phase involving neutral molecules and metal ions.^10,11^ Such an isolated and unperturbed environment has proven as a powerful tool to study the intrinsic properties of the ‘active site’ of a catalyst.^12^

Interactions between metal atoms and molecules are important in many disciplines of chemistry, such as coordination chemistry,^13^ organometallic chemistry^14^ and catalysis.^15–17^ Computational chemistry, as a powerful tool to investigate such interactions, is generally used to calculate structures and energetics of metal-containing complexes, and can reveal how the strength of chemical bonds is influenced by metal atoms.^18^ If the cleavage of a specific chemical bond is important for the reaction, weakening of this bond by a metal atom will result in a lower activation energy and thus manifest robust catalytic activity.

In this work we chose diol molecules (1,6-hexanediol and 1,8-ocatanediol) and Au complexes as exemplar systems to illustrate such a concept at the molecular level. A key advancement in this work is to use superfluid helium nanodroplets^19,20^ as nano-reactors to form and isolate diol–Au_*n*_ clusters (*n* = 1, 2, …), *i.e*., by the sequential addition of diol molecules and Au atoms to helium nanodroplets (Fig. S1†). The droplets were then ionized by electron impact (100 eV). The electron initially can produce a He^+^ ion near the surface of the droplet,^22^ which is mobile on account of resonant charge hopping and can transfer its charge to the embedded complexes and create ionized complexes.^23^ An alternative route of ionization is *via* electronically excited He* (2^3^S) atoms,^24^ which can also occur given the relatively small droplet sizes in this work. The resulting diol–Au_*n*_ cations subsequently undergo chemistry and any ions ejected into the gas phase were then detected by mass spectrometry. By this route any surface contact was avoided, allowing the effect of Au atoms on the dissociative ionization reactions of diol molecules to be unambiguously identified.

## Results and discussion

By careful control of the partial pressure of diol molecules using a needle valve and the vapour pressure of Au by the oven temperature, multiple pickup of diol molecules was minimized by reducing the diol–H^+^ channel, which is a signature of diol clusters, and on average each helium droplet contained one Au atom (Fig. S2†). This is essential to maximize the proportion of helium droplets containing one Au atom and illustrate the effect of a single Au atom on the dissociation chemistry of the diol–Au cation. As the pickup follows Poisson statistics,^19^ under these conditions ∼37% of helium droplets contained no gold atom, ∼37% had only one gold atom, ∼18% had two gold atoms and the remaining ∼8% with three or more gold atoms. Given the low temperature of helium droplets (0.37 K), no reaction between the gold atom(s) and diol molecules is possible prior to ionization so the sequential pickup of diol molecules and Au atoms led to the formation of van der Waals complexes. Both O atoms in diol molecules can serve as binding sites for Au atoms; so the complexes may have different configurations when helium droplets pick up more than one Au atom. For example, Au–HOC_6_H_12_OH–Au and HOC_6_H_12_OH–Au_2_ can be formed at a 1 : 1 ratio when 2 Au atoms are co-added to helium nanodroplets. As detailed later, the effect of Au atoms to the C–O and C–C bonds within the diol–Au_2_ cation is similar to that of a single Au atom, suggesting that multiple pickup of Au does not complicate the reactions.

**Fig. 1:**
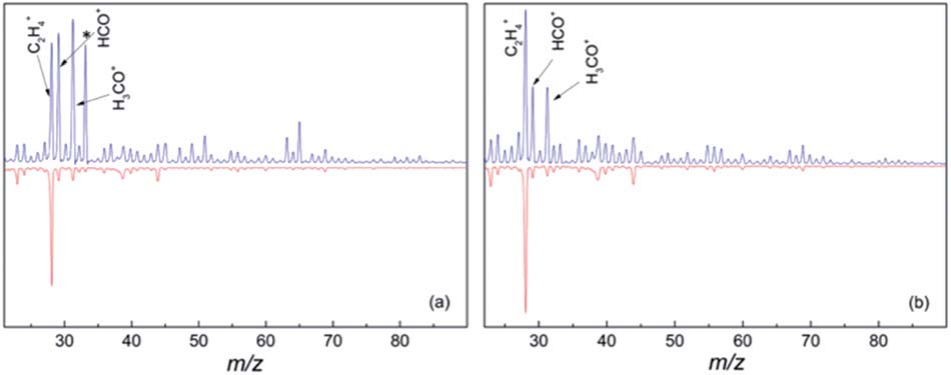
Mass spectra of diols (upper panels) and diol–Au_*n*_ complexes (lower panels). (a) Mass spectra of 1,6-hexanediol and the 1,6-hexanediol–Au_*n*_ complex; (b) mass spectra of 1,8-octanediol and the 1,8-octanediol–Au_*n*_ complex. The helium droplet background ion signal was subtracted in all of the spectra. The prominent ions correspond to peaks at *m*/*z* 28, 29 and 31, which are assigned to C_2_H_4_^+^, HCO^+^ and CH_2_OH^+^, respectively. The asterisk in (a) marks the O_2_H^+^ peak at *m*/*z* 33, which is likely due to trace O_2_ in the hexanediol sample owing to incomplete degassing. Excluding this product, C_2_H_4_^+^ accounts for 17% of the overall signal in the hexanediol mass spectrum, which increases to 66% when Au is co-added. For octanediol and octanediol–Au complexes, the *m*/*z* 33 peak is absent and the C_2_H_4_^+^ signal accounts for 26% and 68% of the overall ion products, respectively.

In the gas phase mass spectrum of the isolated diol molecules the prominent ions are seen at *m*/*z* 31, 41, 42 and 67 for 1,6-hexane diol, and *m*/*z* 31, 41, 55, 67, 82 for 1,8-octanediol.^21^ However, the mass spectra in helium droplets are very different. As seen in Fig. 1, C_2_H_4_^+^, HCO^+^ and CH_2_OH^+^ are the major products, accounting for 17%, 19% and 21% in the mass spectrum of hexanediol-doped helium droplets, and 26%, 13% and 13% for octanediol-doped helium droplets, respectively. In the mass spectra of diol–Au complexes, C_2_H_4_^+^ is the sole prominent product, accounting for ∼66% of the overall ion in the mass spectrum of hexanediol–Au complex and ∼68% for the octanediol–Au complex, respectively. Meanwhile, both HCO^+^ and CH_2_OH^+^ signals are drastically reduced, *i.e.*, to ∼8% in both cases for hexanediol–Au, and ∼5% in both cases for octanediol–Au. When considering the contribution from the droplets that contain no gold atom (∼37%), the abundance of C_2_H_4_^+^ ion is calculated as 95% for hexanediol–Au and 92% for octanediol–Au. This remarkable change of chemical outcome suggests a pronounced catalytic effect of gold atoms on the ionization-induced chemistry. Unlike single-atom catalysis by metal ions,^10,11^ the fragments detected in the low-mass region result from the dissociation of diol cations. This observation is taken as evidence that the reactions were catalyzed by neutral Au atoms.

**Fig. 2:**
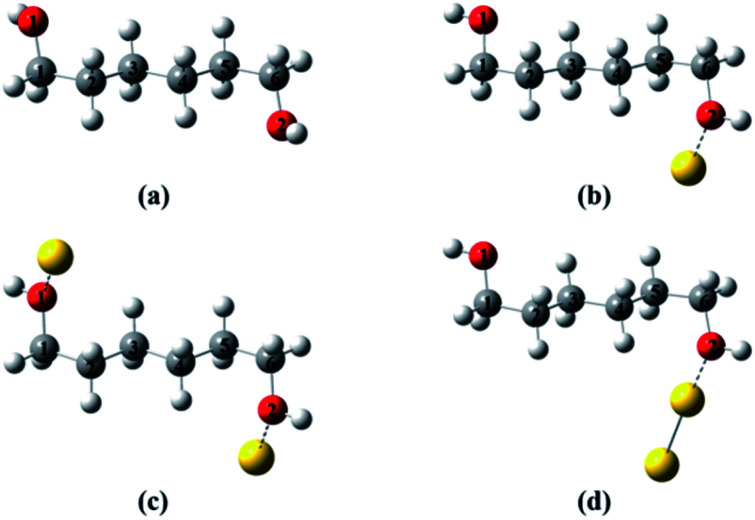
Lowest-energy structures of hexanediol–Au complex cations. (a) Hexanediol cation; (b) hexanediol–Au cation; (c) Au–hexanediol–Au cation; (d) hexanediol–Au_2_ cation. The geometry optimization starts with neutral 1,6-hexanediol, yielding a structure similar to that obtained by Chen *et al.*^25^ This was then used to construct initial configurations in geometry optimizations for hexanediol–Au, Au–hexanediol–Au and hexanediol–Au_2_. The optimized geometries of the neutrals were used as initial structures of the cations in the calculations. The labelling of atoms is used in Fig. 3.

**Fig. 3:**
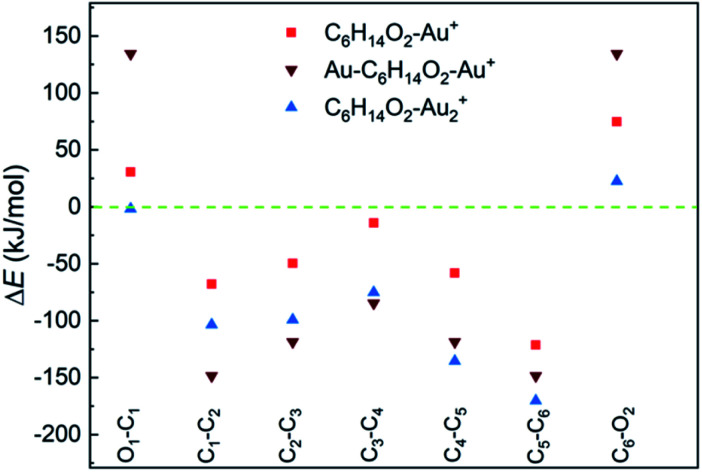
Changes of C–C and C–O bond energies in 1,6-hexanediol–Au_*n*_ cations (*n* = 1 and 2) relative to the 1,6-hexanediol cation. All of the C–C bonds are strengthened by the Au atom and C–O bonds are generally weakened. The bond energies and the bond energy changes are listed in Table S1.†

To provide a heuristic interpretation we performed density functional theory (DFT) calculations on 1,6-hexanediol, its Au-containing complexes and the corresponding ions, with the focus on the C–C and C–O bonds in hexanediol, the fissions of which account for major fragments in the mass spectra. The geometry optimizations progressed from neutral 1,6-hexanediol, hexanediol–Au, Au–hexanediol–Au and hexanediol–Au_2_ complexes, and the optimized structures of neutrals were then used as the initial configurations for the geometric optimization of the corresponding ions (Fig. 3†). This allowed accurate determination of low-energy structures without a complete structural search (1,6-hexanediol is known to have over 300 conformers^25^). The lowest energy structures of the neutral molecules and ions are shown in Fig. 2 and the C–C and C–O bond energies are summarized in Table S1†. Note that 1,8-ocatanediol and its Au-containing complexes were not computed due to the similarity to 1,6-hexanediol and the 1,6-hexanediol–Au_*n*_ complexes.

DFT calculations suggest a weak interaction between neutral 1,6-hexanediol and Au atoms (with a binding energy < 2 kJ mol^−1^, Table S2†) and a much stronger interaction in 1,6-hexanediol–Au cation (with a binding energy of 157 kJ mol^−1^), which has major effect on the C–C and C–O bond energies in the hexanediol–Au_*n*_^+^ complex (see Fig. 3). Both C–O bonds have a bond energy of 400 kJ mol^−1^ in the isolated hexanediol cation, which are stronger than all of the C–C bonds (Table S1†). However, when a gold atom is attached, both C–O bonds are significantly weakened, *i.e*., by 75 kJ mol^−1^ for the nearby C–O bond (where the gold atom is attached) and 31 kJ mol^−1^ for the remote C–O bond. Meanwhile, all of the C–C bonds are significantly strengthened. The most significant increases of bond energies occur in the C–C bonds next to the O atoms, *i.e*., by 121 kJ mol^−1^ and 68 kJ mol^−1^, respectively; while the least increase arises in the middle C–C bond (by 14 kJ mol^−1^). For the Au–hexanediol–Au cation, the degrees of weakening of the C–O bonds and strengthening of the C–C bonds by the Au atoms is even higher: both C–O bonds are weakened by 134 kJ mol^−1^ and the increase of the C–C bond energies ranges from 85 to 148 kJ mol^−1^. For the hexanediol–Au_2_ cation, the changes in C–O bond strengths are relatively minor but all of the C–C bonds have similar strengths to the Au–hexanediol–Au cation, with the nearby C–C bond strengthened by 170 kJ mol^−1^. Similar effects may occur in diol–Au complexes containing 3 or more Au atoms, which accounts for ∼8% of the total diol–Au complexes by the pickup statistics.

Our calculations provide strong support for the experimental observations. For the 1,6-hexanediol cation, both C–O bonds are stronger than any of the C–C bonds within the molecule so the cleavage of C–O bonds are less favoured compared with the C–C bonds. As a result, the O-containing fragments are prominent in the mass spectrum of hexanediol. In contrast, when Au atoms are co-added, the C–O bonds are generally weakened (with exception of the remote C–O bond in hexanediol–Au_2_) and all of the C–C bonds are significantly strengthened. Consequently, the cleavage of C–O bonds is easier and the fission of C–C bonds becomes more difficult when compared with 1,6-hexanediol. Remarkably, both C–O bonds are considerably weaker than all of the C–C bonds in these Au-containing complexes, which will make the cleavage of the C–O bonds favoured in ion–molecule reactions. The loss of OH groups lead to the formation of the C_6_H_12_^+^ ion, which subsequently dissociates with C_2_H_4_^+^ as the preferential product (Fig. S4†).

## Conclusions

In closing, we report a striking observation that a single metal atom weakly interacting with molecules in a van der Waals complex can nevertheless serve as a single-atom catalyst in ionization-induced dissociative reactions. DFT calculations showed that a gold atom significantly alters the bond energies in the 1,6-hexanediol cation, *i.e.*, weakening of the two C–O bonds and strengthening all of the C–C bonds, making both C–O bonds significantly weaker than all of the C–C bonds. Consequently, the cleavage of C–O bonds becomes favourable in the dissociation of diol–Au cations and the C_2_H_4_^+^ ion becomes the prominent product. This is a molecular-level showcase of the Arrhenius law, where lowering the activation energy plays a paramount role in the reactions. Note that the charge transfer ionization by He^+^ deposits excess energy into the diol–Au complexes (>10 eV), which may complicate the reaction mechanism. Potentially, this can be resolved by using a lower energy ionization technique, *e.g.*, photoionization. Alternatively, it might be worth trying to study Au–diol complex cations in the solution phase, *e.g*., by adding Au salt to diol solutions, where the Au^+^ complexing with diol molecules can weaken the C–O bonds and strengthen all of the C–C bonds, and hence can catalyse C–O bond cleavage reactions. In this context, this work suggests a new strategy in catalysis, *i.e*., by preparation of molecular complex precursors where specific chemical bonds important for chemical reactions are pre-activated *via* complexing. More broadly, the discovery in this work will encourage theoreticians to study how chemical bonds are influenced by atomic catalysts in numerous reactions, which will lead to a much better understanding of catalysis, and ultimately, to the rational design of catalysts.

## Author contributions

SY led the research. AMAH and BS ran the experiments and analysed experimental data. AME and JY contributed to discussions and the manuscript preparation. HW and QL performed all of the DFT calculations.

## Conflicts of interest

There are no conflicts to declare.

## Supplementary Material

SC-011-D0SC03523H-s001
