# Supplementary material for: Ion-molecule reactions catalyzed by a single gold atom
Source: Chem Sci. 2020 Jul 27;11(32):8502–5. doi: 10.1039/d0sc03523h (PMC8163403; doi:10.1039/d0sc03523h)
Supplement: SC-011-D0SC03523H-s001 [file SC-011-D0SC03523H-s001.pdf]

Electronic Supplementary Material (ESI) for Chemical Science.
This journal is © The Royal Society of Chemistry 2020

Ion-molecule Reactions Catalyzed by a Single Gold Atom

Shengfu Yang,[a*] Hong Wu,[b] Qiquan Luo,[b] Aula Al Hindawi,[ac] Berlian Sitorus,[ad] Andrew M. Ellis[a] and Jinlong Yang[b*]

[a] School of Chemistry, University of Leicester, Leicester LE1 7RH, UK

[b] Hefei National Laboratory for Physical Sciences at the Microscale, University of Science and Technology of China, Hefei 230026, P.R. China.

[c] Department of Chemistry, College of Education for Pure Science, University of Karbala, Kerbala, Iraq

[d] Department of Chemistry, Tanjungpura University, Pontianak, Indonesia

* Corresponding Authors. Email addresses:sfy1@le.ac.uk; jlyang@ustc.edu.cn.

Telephone +44 (0)116 252 2127; Fax +44 (0)116 252 3789.

1. Experimental Section

1.1 The apparatus and experimental conditions

An ultra-high vacuum helium nanodroplet source was used to form diol-Au$_n$ complexes (n = 1, 2, 3 …) and to record their mass spectra (see supplementary Fig. 1). Briefly, helium nanodroplets were generated by supersonic expansion of pressurized cold helium gas through a 5 µm pinhole nozzle, which were then skimmed into a collimated droplet beam prior to entering the pickup region. The helium stagnation pressure was 15 bar and the nozzle temperature was 11 K, producing helium nanodroplets consisting of ~5 × 10^3 atoms on average.[1] In the pickup region helium nanodroplets first entered a diol pickup cell and then travelled to a gold evaporator. 1,6-hexanediol and 1,8-octanediol were purchased from Sigma-Aldrich with stated purities of 99% and 98%, respectively. The diol samples were heated to 35-50 °C in order to remove air contamination prior to exposing the vapor to the helium droplet beam, and the doping rate was controlled by use of a needle valve. To add Au to helium droplets, high-purity gold (>99.95%) was resistively heated to 1260 K in an oven to produce single gold atoms in the gas phase. Mass spectrometry suggested that on average each helium droplet picked up only one gold atom (see Supplementary Fig. 2).

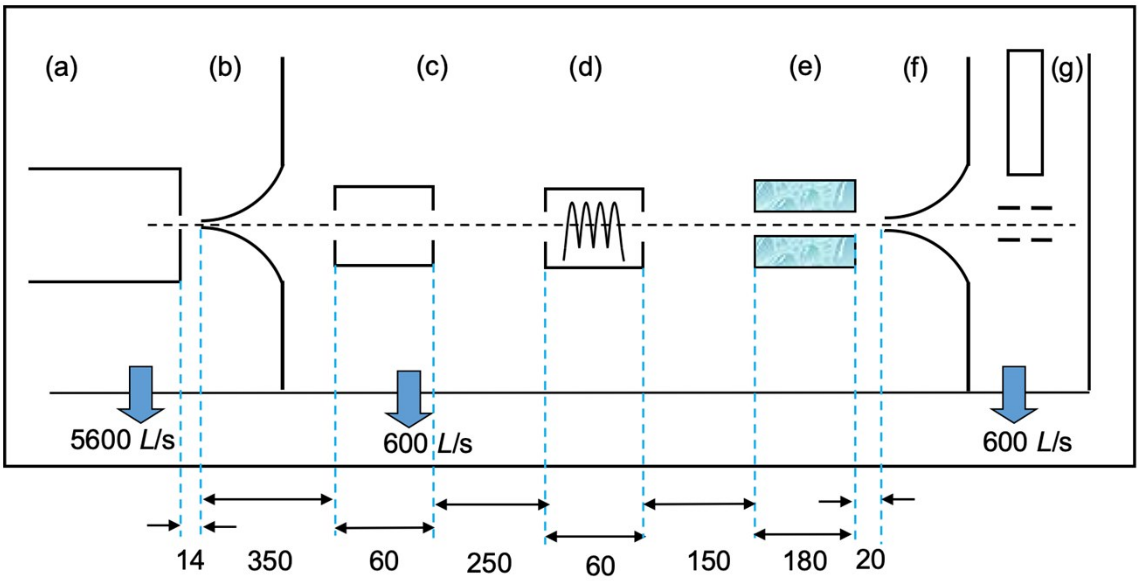

Supplementary Fig. 1 Schematic diagram of the experimental apparatus. (a) Continuous helium droplet source; (b) 0.5 mm skimmer; (c) Gas cell; (d) Evaporator of gold; (e) liquid

nitrogen cold trap; (f) 2 mm skimmer; (g) quadrupole mass spectrometer (QMS). The distances between the vacuum units are in millimeters.

As multiple pickup of diol molecules in helium droplets will complicate the reaction dynamics, we carefully controlled the doping of diol molecules by a needle valve to avoid this. We focused particularly on the diol-H^+ signal in the mass spectra, which can only be formed via multiple pickup events. By monitoring this specific channel while adjusting the needle valve, the partial pressure of the diol dopant was reduced until this signal fell into the noise level. We also noticed that at this doping level, the mass spectrum showed no diol signals without helium droplets (blocked by a gate-valve after the first skimmer). This also excludes possible influence by the diol molecules drifting into the Au oven, which might fragment at the oven temperature. Note that the absolute fraction of droplets doped with single diol molecules does not influence the experimental observation because those empty droplets have no contribution to the mass spectrum of diol molecules and the diol-Au_n complexes.

The focus of this work is to study how a single Au atom affects the ion-molecule reactions of diol molecules; so we deliberately maximized the fraction of helium droplets containing only one Au atom by using the single pickup condition. Indeed, multiple pickup of Au atoms was inevitable due to the statistic nature of the pickup process, which may influence the dissociation pathways and complicate the interpretation. However, density functional theory (DFT) calculations showed similar effect to the C-C and C-O bonds in Au-1,6-hexanediol-Au and 1,6-hexanediol-Au_2 cations when compared to that of 1,6-hexanediol-Au (see th main text), suggesting that multiple pickup events do not modify the fragmentation pathways. We expect the similar trend for larger clusters containing three or more Au atoms, which, however, should have little effect in this work due to the low abundance (8%).

Four sets of mass spectra were recorded for hexanediol and octanediol, respectively, following the order of pure helium droplets, the addition of diol molecules to helium droplets, the co-addition of Au atoms to helium droplets and closure of the diol inlet. The temperature of Au oven was maintained at 1100 K when recoding mass spectra of diol molecules, at which no Au was seen in the mass spectra. The doping rate of diol molecules was then maintained when the Au oven was turned on to form diol-Au_n complexes, and the mass spectrum of pure Au clusters was obtained by the closure of gas needle valve. In each step only one experimental condition was changed, ensuring that the helium droplet background can be subtracted and the changes in mass spectra in each step can be identified.

1.2 Establishing the single pickup conditions for Au atoms

The number of Au atoms in helium droplets follows a Poisson distribution, *i.e.*,

$$P_k(z) = \frac{z^k e^{-z}}{k!} \quad [1]$$

where k is the number of Au atoms in a helium droplet, z is the pickup parameter defined by $z = \sigma n l$. Here, σ is the geometric cross section of the helium droplets, n is the number density of Au atoms in the oven evaporator, which depends on the oven temperature, and l is the length of the oven. The quantity z is the number of collisions when a droplet with a geometric cross section of σ passes through the oven. Assuming a near unity pickup probability when helium droplets collide with molecules/atoms, z =1 is referred as the single pickup condition.

According to Eq. [1], the maximum value of P_1, the fraction of helium droplets containing only one Au atom, is achieved with z = 1, which is referred as single pickup condition here. Under this condition, the proportions of helium droplets containing no Au atom, one Au atom and two Au atoms are P_0 = 36.8%, P_1 = 36.8% and P_2 = 18.4%, respectively. The remaining droplets containing more than two Au atoms account for the rest 8%. The Au^+ and Au_2^+ ratio is therefore expected to be 2:1 ratio in the mass spectrum of Au clusters when no diol molecule is co-added – this was the experimental condition we achieved in order to fulfil the single pickup condition. We therefore carefully optimized the temperature of the Au evaporator and recorded the mass spectrum with only Au added to the helium droplets in both set of experiments to arrive at this ratio.

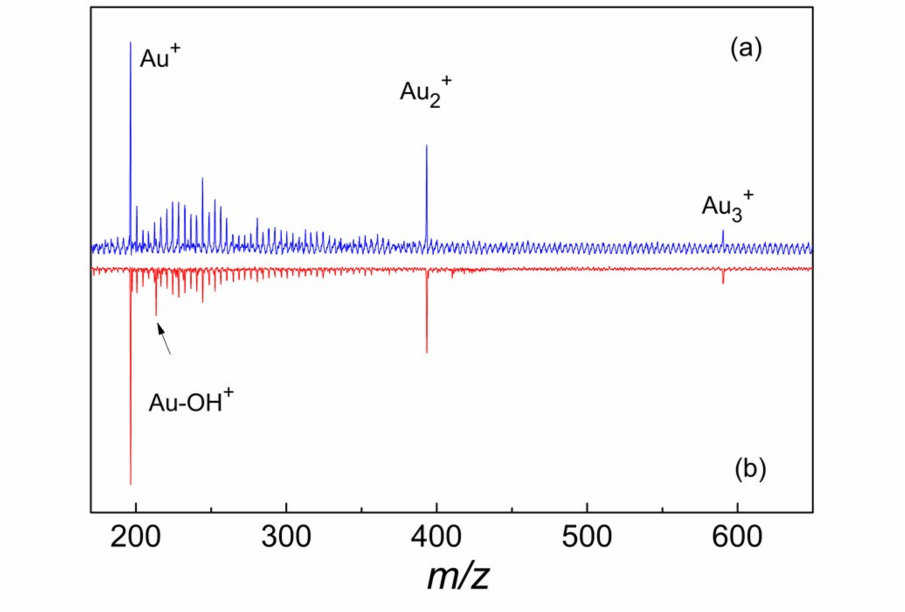

Supplementary Fig. 2 Mass spectra of (a) Au clusters and (b) 1,6-hexanediol-Au complexes in the mass range of 170 – 650 amu. For pure Au clusters, the ratio of Au^+/Au_2^+ is about ~2:1, indicating on average one Au atom was added to helium droplets. When Au is co-added to helium nanodroplets with 1,6-hexanediol, the relative abundance of Au_2^+ slightly decreases and the same does the Au_3^+. This is because when two Au atoms entered helium droplets, they had equal probability to attach to the two O atoms of the diol molecule and form either $HOC_6H_{12}OH$-Au_2 or Au-$HOC_6H_{12}OH$-Au. The dissociation of latter is more likely to produce Au^+ when compared with $HOC_6H_{12}OH$-Au_2. The lower panel also show minor ion products with OH attached to Au, which only is ~2% of the $C_2H_4^+$ signal.

1.3 Calculation of the abundance of $C_2H_4^+$

When Au is co-added to helium droplets with a diol molecule under single pickup conditions, we expect 36.8% helium droplets to contain no Au atoms and thus 63.2% signal in the mass spectra will be originated from the diol-Au_n complexes formed in helium droplets. For 1,6-hexanediol and 1,8-octanediol, the helium droplets containing no Au atom will also contribute to the mass spectra. Assuming that the abundance of the $C_2H_4^+$ ion in mass spectra without the addition of Au is x and the abundance of the $C_2H_4^+$ ion in mass spectra of diol-Au_n complexes is y, the contribution of the droplets containing no Au to the $C_2H_4^+$ ion is 0.368 x,

which needs to be subtracted. Therefore, the abundance of the $C_2H_4^+$ product in the mass spectra of helium droplets by diol-Au$_n$ clusters can be calculated as:

$$A = \frac{y - 0.368\,x}{0.632} \qquad [2]$$

By using the Eq.[2], the abundance of the $C_2H_4^+$ ion with the addition of Au atoms can be calculated as 94.6% for hexanediol-Au$_n$ and 92.2% for octanediol-Au$_n$.

2. Quantum chemical computation methods and results

2.1 Computational methods

All of the calculations are performed using density functional theory (DFT) implemented in the Gaussian 09 program package using the B3LYP-D3 method , which employs an empirical dispersion correction to account for weak interactions.[2] The all-electron 6-311+G(d, p) basis sets for C, O, and H, and the Lanl2dz basis set associated effective core potential (ECP) for gold are used for geometry optimizations. Counterpoise corrections are performed to determine the bond energies, which take into account of basis set superposition errors (BSSE). Harmonic vibrational frequencies are calculated to confirm that the optimized structures are energy minima.

2.2 Low-energy structures of hexanediol-Au, Au-hexanediol-Au and hexanediol-Au$_2$ complexes

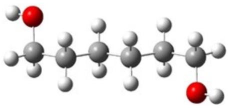

s1
tG-TTTG+t
$\Delta E = 0$

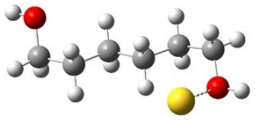

s1/Au-1
tG-TTTG+t(g-)

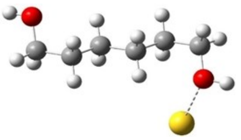

s1/Au-2
tG-TTTG+t(g+)

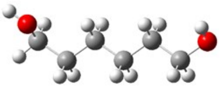

s2
tG-TTTTg+
Δ*E* = 1.01 kJ/mol

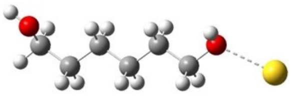

s2/Au-1
tG-TTTTg+(t)

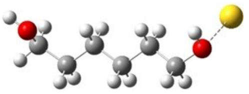

s2/Au-2
tG-TTTTg+(g-)

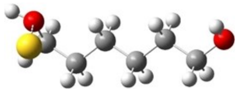

s2/Au-3
(g-)tG-TTTTg+

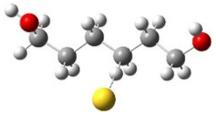

s2/Au-4

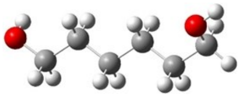

s3
g+TTTTG+t
Δ*E* = 1.11 kJ/mol

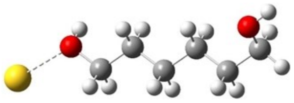

s3/Au-1
(t)g+TTTTG+t

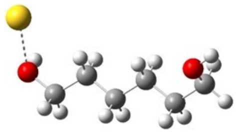

s3/Au-2
(g-)g+TTTTG+t

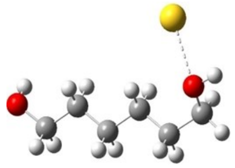

s3/Au-3
g+TTTTG+t(g-)

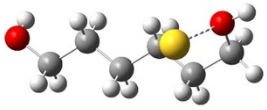

s3/Au-4
g+TTTTG+t(g+)

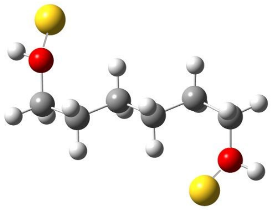

s1/2Au-1
(g-)tG-TTTG+t(g+)
-44° 33°

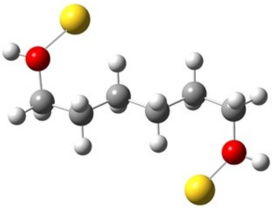

s1/2Au-2
(g+)tG-TTTG+t(g+)
86° 34°

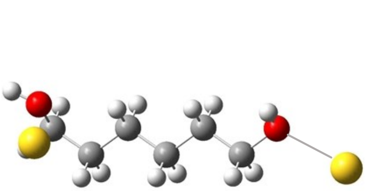

s2/2Au-1
(g-)tG-TTTTg+(t)
-45° -178°

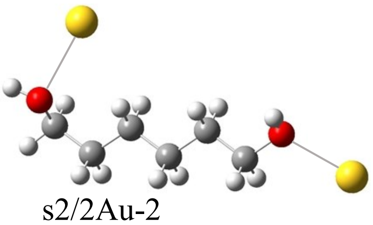

s2/2Au-2
(g+)tG-TTTTg+(t)
-89° -175°

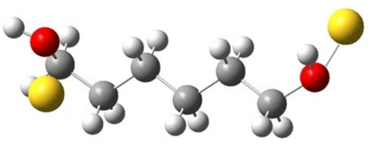

s2/2Au-3
(g-)tG-TTTTg+(g-)
-41° -60°

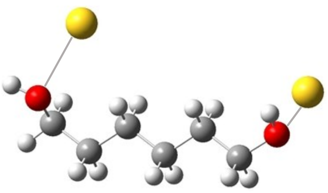

s2/2Au-4
(g+)tG-TTTTg+(g-)
86° -62°

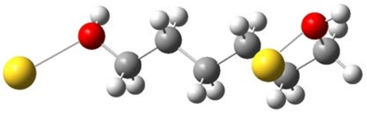

s3/2Au-1
(t)g+TTTTT+t(g+)
178° 46°

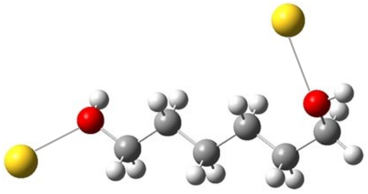

s3/2Au-2
(t)g+TTTTT+t(g-)
176° -90°

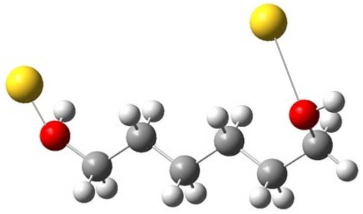

s3/2Au-3
(g-)g+TTTTT+t(g-)
176° -90°

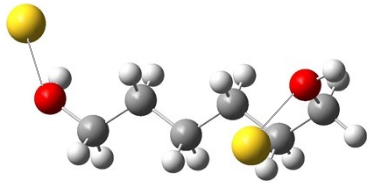

s3/2Au-4
(g-)g+TTTTT+t(g+)
-51° 49°

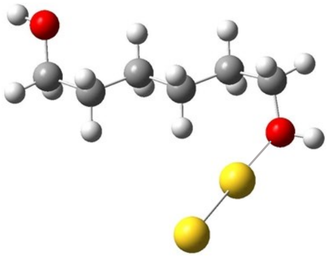

s1/Au_2-1
tG-TTTG+t(g'-)
-82°

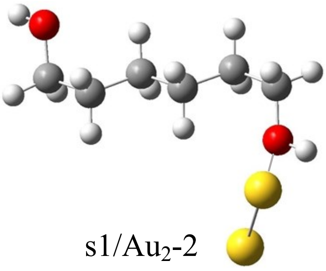

s1/Au_2-2
tG-TTTG+t(g'+)
39°

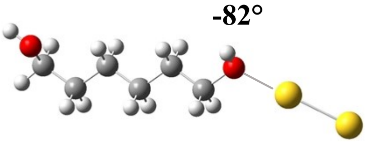

s2/Au_2-1
tG-TTTTg+(t')
-169°

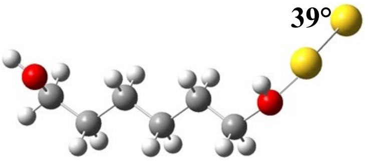

s2/Au_2-2
tG-TTTTg+(g'-)
-66°

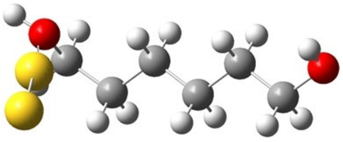

s2/Au_2-3
(g'-)tG-TTTTg+
-40°

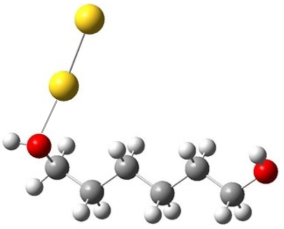

s2/Au_2-4
(g'+)tG-TTTTg+
84°

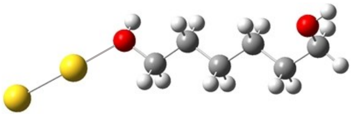

s3/Au_2-1
(t')g+TTTTG+t
178°

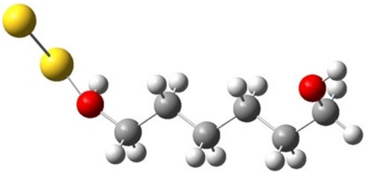

s3/Au_2-2
(g'-)g+TTTTG+t
-64°

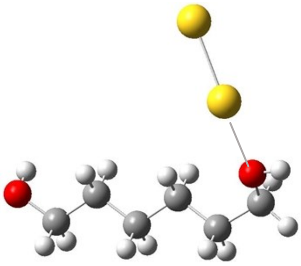

s3/Au_2-3
g+TTTTG+t(g'-)
-86°

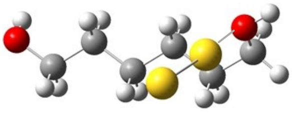

s3/Au_2-4
g+TTTTG+t(g'+)
42°

Supplementary Fig. 3 Low-energy structures of hexanediol-Au, Au-hexanediol-Au and hexanediol-Au_2 complexes calculated using B3LYP-D3/6-311+G(d,p) method. The three low-energy structures of 1,6-hexanediol are s1, s2 and s3, respectively, with s1 having the lowest energy. The relative energies of s2 and s3 are 1.01 kJ/mol and 1.11 kJ/mol, respectively. Each torsion angle can have three possible torsional states, with approximately 180º, 60º, and -60º for trans, gauche+, and gauche– conformations, respectively. The letter codes are denoted by t, g+, or g- for hydroxyl groups, T, G+, or G- for the aliphatic chain, and (t), (g+), or (g-) for O-Au, respectively. H atom in white, C atom in gray, O atom in red and Au atom in gold, respectively.

2.3 Relative electronic energies

Supplementary Table 1 Bond energies of the C-C and C-O bonds in the cations of hexanediol and its Au-containing complexes, and the changes in bond strengths due to Au atom(s). All units are in kJ/mol.

Bond	E_1 [a]	E_2 [b]	E_3 [c]	E_4 [d]	ΔE_2 [e]	ΔE_3 [f]	ΔE_4 [g]

O_1-C_1	-400.2	-369.6	-265.8	-402.0	+30.6	+134.3	-1.8
C_1-C_2	-320.6	-388.4	-468.9	-424.1	-67.8	-148.4	-103.5
C_2-C_3	-344.1	-393.7	-462.7	-443.2	-49.6	-118.6	-99.1
C_3-C_4	-380.8	-395.0	-465.3	-455.9	-14.2	-84.6	-75.1
C_4-C_5	-344.1	-402.1	-462.7	-479.5	-58.0	-118.6	-135.4
C_5-C_6	-320.6	-442.0	-468.9	-490.7	-121.4	-148.4	-170.2
C_6-O_2	-400.2	-325.4	-265.8	-377.6	+74.8	+134.3	+22.6

[a] Bond energies in 1,6-hexanediol cation; [b] bond energies in hexanediol-Au cation; [c] bond energies in Au-hexanediol-Au cation; [d] bond energies in hexanediol-Au_2 cation; [e] $\Delta E_2 = E_2 - E_1$; [f] $\Delta E_3 = E_3 - E_1$; [g] $\Delta E_4 = E_4 - E_1$.

Supplementary Table 2 Relative electronic energies for 1,6-hexanediol-Au complexes (***E***) and the binding energies between 1,6-hexanediol and gold atom. All units are in kJ/mol. The lowest energy structure is s1/Au-2,with a binding energy as low as 1.87 kJ/mol between the hexanediol molecule and the gold atom.

**Structures**	***E***	**Δ*E* (Au-O)**
**s1/Au-1**	0.45	2.32
**s1/Au-2**	0.00	1.87
**s2/Au-1**	4.64	5.50
**s2/Au-2**	1.11	1.98
**s2/Au-3**	0.08	0.94
**s2/Au-4**	3.28	4.14
**s3/Au-1**	4.79	5.55
**s3/Au-2**	3.33	4.09
**s3/Au-3**	4.24	5.00
**s3/Au-4**	0.73	1.49

Supplementary Table 3 Relative electronic energies of Au-hexanediol-Au complexes complexes. All units are in kJ/mol.

**Au-1,6-hexanediol-Au**	**Relative Energy**
**s1/2Au-1**	0.00
**s1/2Au-2**	8.55
**s2/2Au-1**	6.89
**s2/2Au-2**	8.86
**s2/2Au-3**	3.26
**s2/2Au-4**	5.58
**s3/2Au-1**	6.99
**s3/2Au-2**	9.07
**s3/2Au-3**	4.85
**s3/2Au-4**	2.91

Supplementary Table 4 Relative electronic energies of hexanediol-Au_2 complexes. All units are in kJ/mol.

**1,6-hexanediol-Au_2**	**Relative Energy**
**s1/Au_2-1**	3.18
**s1/Au_2-2**	0.00
**s1/Au_2-3**	0.05
**s2/Au_2-1**	8.86
**s2/Au_2-2**	5.98
**s2/Au_2-3**	1.86
**s2/Au_2-4**	5.87
**s3/Au_2-1**	9.46
**s3/Au_2-2**	5.17
**s3/Au_2-3**	7.37
**s3/Au_2-4**	1.94

3. Proposed reaction pathways for the preferential formation of $C_2H_4^+$

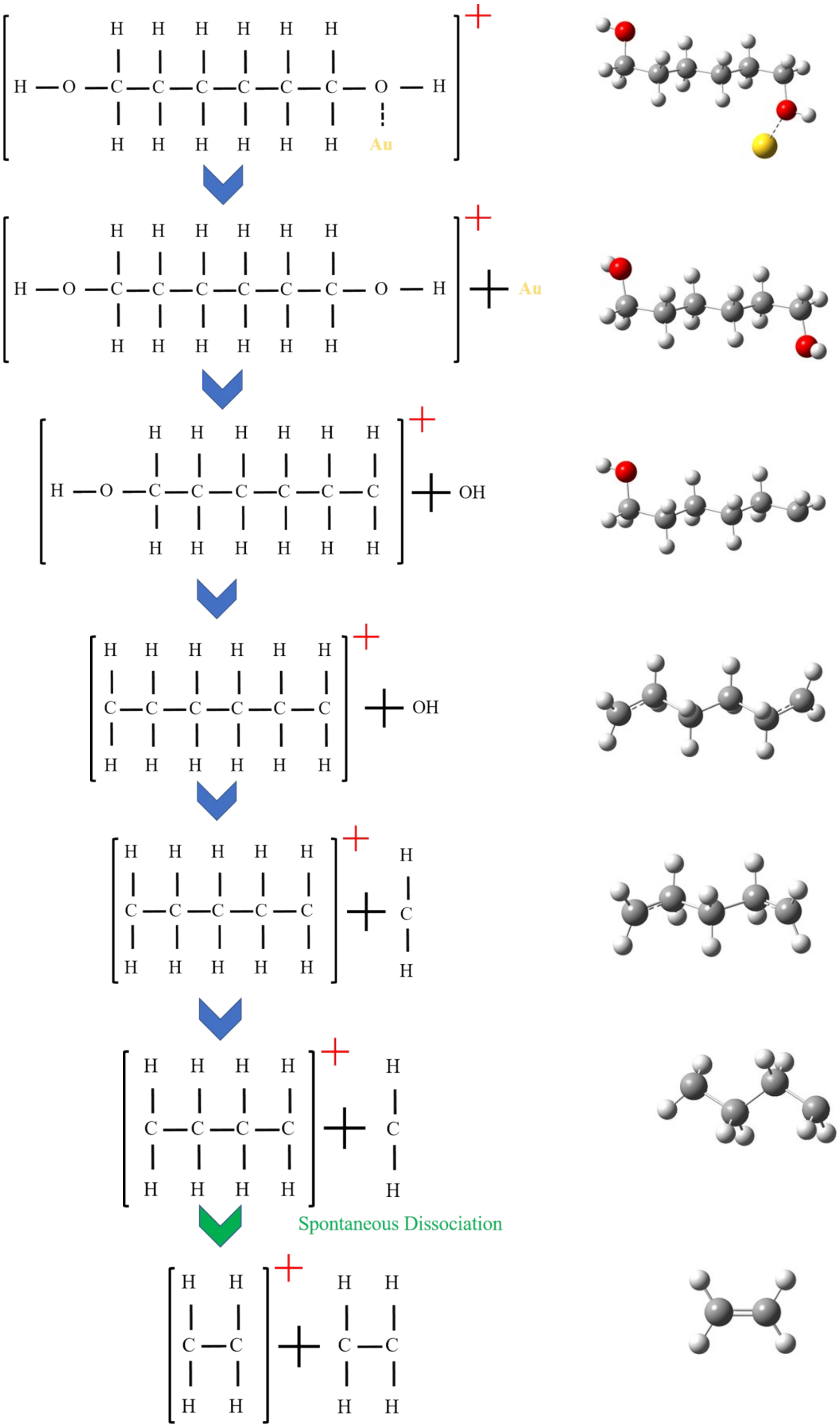

Supplementary Fig. 4 Illustrative cartoon for the preferential formation of $C_2H_4^+$ in the dissociation of 1,6-hexandiol-Au cation. The dissociation pathway is proposed following the

order of bond energies in 1,6-hexanediol-Au cation. The $C_4H_8^+$ ion can undergo self-dissociation, which leads to the formation of $C_2H_4^+$.

References

1. A. V. Kanaev, L. Museur, T. Laarman, M. C. Castex, S. Monticone, K. von Haeften and T. Möller, *J. Chem. Phys.* **2001**, *115*, 10248-10253.
2. S. Grimme, J. Antony, S. Ehrlich, H. Krieg, *J. Chem. Phys.* **2010**, *132*, 154104.
